# FieldSAFE: Dataset for Obstacle Detection in Agriculture

**DOI:** 10.3390/s17112579

**Published:** 2017-11-09

**Authors:** Mikkel Fly Kragh, Peter Christiansen, Morten Stigaard Laursen, Morten Larsen, Kim Arild Steen, Ole Green, Henrik Karstoft, Rasmus Nyholm Jørgensen

**Affiliations:** 1 Department of Engineering, Aarhus University, Aarhus N 8200, Denmark; repetepc@gmail.com (P.C.); msl@eng.au.dk (M.S.L.); hka@eng.au.dk (H.K.); rnj@eng.au.dk (R.N.J.); 2 Conpleks Innovation ApS, Struer 7600, Denmark; morten.larsen@conpleks.com; 3 AgroIntelli, Aarhus N 8200, Denmark; kas@agrointelli.com (K.A.S.); olg@agrointelli.com (O.G.)

**Keywords:** dataset, agriculture, obstacle detection, computer vision, cameras, stereo imaging, thermal imaging, LiDAR, radar, object tracking

## Abstract

In this paper, we present a multi-modal dataset for obstacle detection in agriculture. The dataset comprises approximately 2 h of raw sensor data from a tractor-mounted sensor system in a grass mowing scenario in Denmark, October 2016. Sensing modalities include stereo camera, thermal camera, web camera, 360${}^{\circ }$ camera, LiDAR and radar, while precise localization is available from fused IMU and GNSS. Both static and moving obstacles are present, including humans, mannequin dolls, rocks, barrels, buildings, vehicles and vegetation. All obstacles have ground truth object labels and geographic coordinates.

## 1. Introduction

For the past few decades, precision agriculture has revolutionized agricultural production systems. Part of the development has focused on robotic automation, to optimize workflow and minimize manual labor. Today, technology is available to automatically steer farming vehicles such as tractors and harvesters along predefined paths using accurate global navigation satellite systems (GNSS) [1]. However, a human operator is still needed to monitor the surroundings and intervene when potential obstacles appear in front of the vehicle to ensure safety.

In order to completely eliminate the need for a human operator, autonomous farming vehicles need to operate both efficiently and safely without any human intervention. A safety system must perform robust obstacle detection and avoidance in real time with high reliability. Additionally, multiple sensing modalities must complement each other in order to handle a wide range of changes in illumination and weather conditions.

A technological advancement like this requires extensive research and experiments to investigate combinations of sensors, detection algorithms and fusion strategies. Currently, a few publicly known commercial R&D projects exist within companies that seek to investigate the concept [2,3,4]. In scientific research, projects investigating autonomous agricultural vehicles and sensor suites have existed since 1997, where a simple vision-based anomaly detector was proposed [5]. Since then, a number of research projects has experimented with obstacle detection and sensor fusion [6,7,8,9,10,11,12,13,14]. However, to our knowledge, no public platforms or datasets are available that address the important issues of multi-modal obstacle detection in an agricultural environment.

Within urban autonomous driving, a number of datasets has recently been made publicly available. Udacity’s Self-Driving Car Engineer Nanodegree program has given rise to multiple challenge datasets including stereo camera, LiDAR and localization data [15,16,17]. A few research institutions such as the University of Surrey [18], Linköping University [19], Oxford [20], and Virginia Tech [21] have published similar datasets. Most of the above cases, however, only address behavioral cloning, such that ground truth data are only available for control actions of the vehicles. No information is thus available for potential obstacles and their location in front of the vehicles.

The KITTI dataset [22], however, addresses these issues with object annotations in both 2D and 3D. Today, it is the de facto standard for benchmarking both single- and multi-modality object detection and recognition systems for autonomous driving. The dataset includes high-resolution grayscale and color stereo cameras, a LiDAR and fused GNSS/IMU sensor data.

Focusing specifically on image data, an even larger selection of datasets is available with annotations of typical object categories such as cars, pedestrians and bicycles. Annotations of cars are often represented by bounding boxes [23,24]. However, pixel-level annotation or semantic segmentation has the advantage of being able to capture all objects, regardless of their shape and orientation. Some of these are synthetically-generated images using computer graphic engines that are automatically annotated [25,26], whereas others are natural images that are manually labeled [27,28].

In agriculture, only a few similar datasets are publicly available. The Marulan Datasets [29] provide multi-sensor data from various rural environments and include a large variety of challenging environmental conditions such as dust, smoke and rain. However, the datasets focus on static environments and only contain a few humans occasionally walking around with no ground truth data available. Recently, the National Robotics Engineering Center (NREC) Agricultural Person-Detection Dataset [30] was made publicly available. It contains labeled image sequences of humans in orange and apple orchards acquired with moving sensing platforms. The dataset is ideal for pushing research on pedestrian detection in agricultural environments, but only includes a single modality (stereo vision). Therefore, a need still exists for an object detection dataset that allows for investigation of sensor combinations, multi-modal detection algorithms and fusion strategies.

While some similarities between autonomous urban driving and autonomous farming are present, essential differences exist. An agricultural environment is often unstructured or semi-structured, whereas urban driving involves planar surfaces, often accompanied by lane lines and traffic signs. Further, distinction between traversable, non-traversable and processable terrain is often necessary in an agricultural context such as grass mowing, weed spraying or harvesting. Here, tall grass or high crops protruding from the ground may actually be traversable and processable, whereas ordinary object categories such as humans, animals and vehicles are not. In urban driving, however, a simplified traversable/non-traversable representation is common, as all protruding objects are typically regarded as obstacles. Therefore, sensing modalities and detection algorithms that work well in urban driving do not necessarily work well in an agricultural setting. Ground plane assumptions common for 3D sensors may break down when applied on rough terrain or high grass. Additionally, vision-based detection algorithms may fail when faced with visual ambiguous information from, e.g., animals that are camouflaged to resemble the appearance of vegetation in a natural environment.

**Figure 1:**
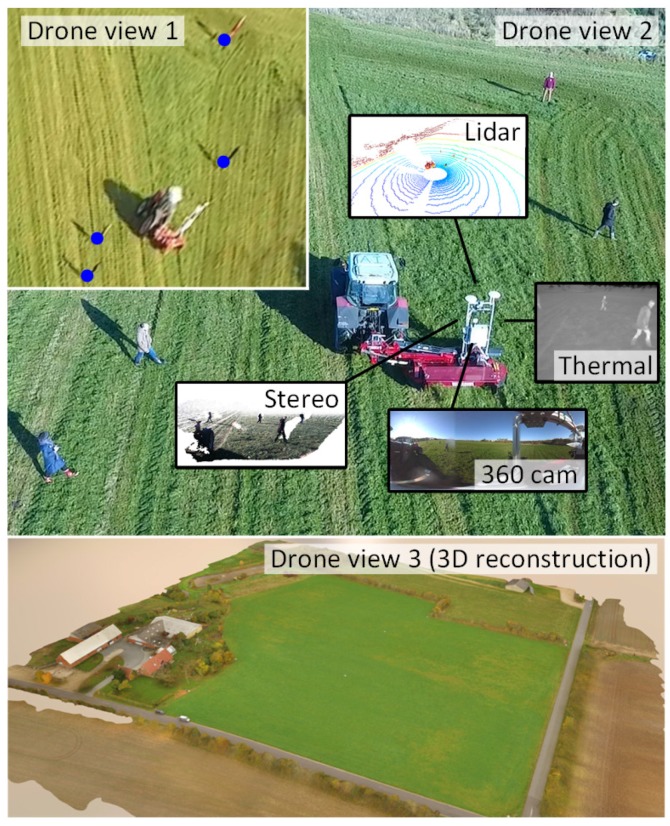
Recording platform surrounded by static and moving obstacles. Multiple drone views record the exact position of obstacles, while the recording platform records local sensor data.

In this paper, we present a flexible, multi-modal sensing platform and a dataset called FieldSAFE for obstacle detection in agriculture. The platform is mounted on a tractor and includes stereo camera, thermal camera, web camera, 360${}^{\circ }$ camera, LiDAR and radar. Precise localization is further available from fused IMU and GNSS. The dataset includes approximately 2 h of recordings from a grass mowing scenario in Denmark, October 2016. Both static and moving obstacles are present including humans, mannequin dolls, rocks, barrels, buildings, vehicles and vegetation. Ground truth positions of all obstacles were recorded with a drone during operation and have subsequently been manually labeled and synchronized with all sensor data. Figure 1 illustrates an overview of the dataset including recording platform, available sensors, and ground truth data obtained from drone recordings. Table 1 compares our proposed dataset to existing datasets in robotics and agriculture. The dataset supports research into object detection and classification, object tracking, sensor fusion, localization and mapping. It can be downloaded from https://vision.eng.au.dk/fieldsafe/.

## 2. Sensor Setup

**Figure 2:**
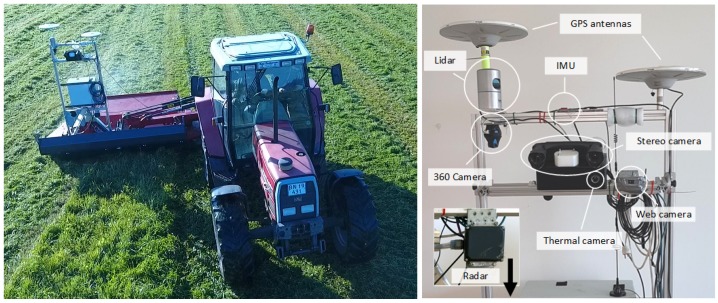
Recording platform.

Figure 2 shows the recording platform mounted on a tractor during grass mowing. The platform was mounted on an A-frame (standard in agriculture) with dampers for absorbing internal engine vibrations from the vehicle. The platform consists of the exteroceptive sensors listed in Table 2, the proprioceptive sensors listed in Table 3 and a Conpleks Robotech Controller 701 used for data collection with the Robot Operating System (ROS) [31]. The stereo camera provides a timestamped left (color) and right (grayscale) raw and rectified image pair along with an on-device calculated depth image. Post-processing methods are further available for generating colored 3D point clouds. The web camera and 360${}^{\circ }$ camera provide timestamped compressed color images. The thermal camera provides a raw grayscale image that allows for conversion to absolute temperatures. The LiDAR provides raw distance measurements and calibrated reflectivities for each of the 32 laser beams. Post-processing methods are available for generating 3D point clouds. The radar provides raw CAN messages with up to 16 processed radar detections per frame from mid- and long-range modes simultaneously. The radar detections consist of range measurements, azimuth angles and amplitudes. ROS topics and data formats for each sensor are available on the FieldSAFE website. Code examples for data visualization are further available on the corresponding git repository.

The proprioceptive sensors include GPS and IMU. An extended Kalman filter has been setup to provide global localization by fusing GPS and IMU with the robot_localization package [32] available in ROS. The localization code and resulting pose information are available along with the raw localization data.

**Figure 3:**
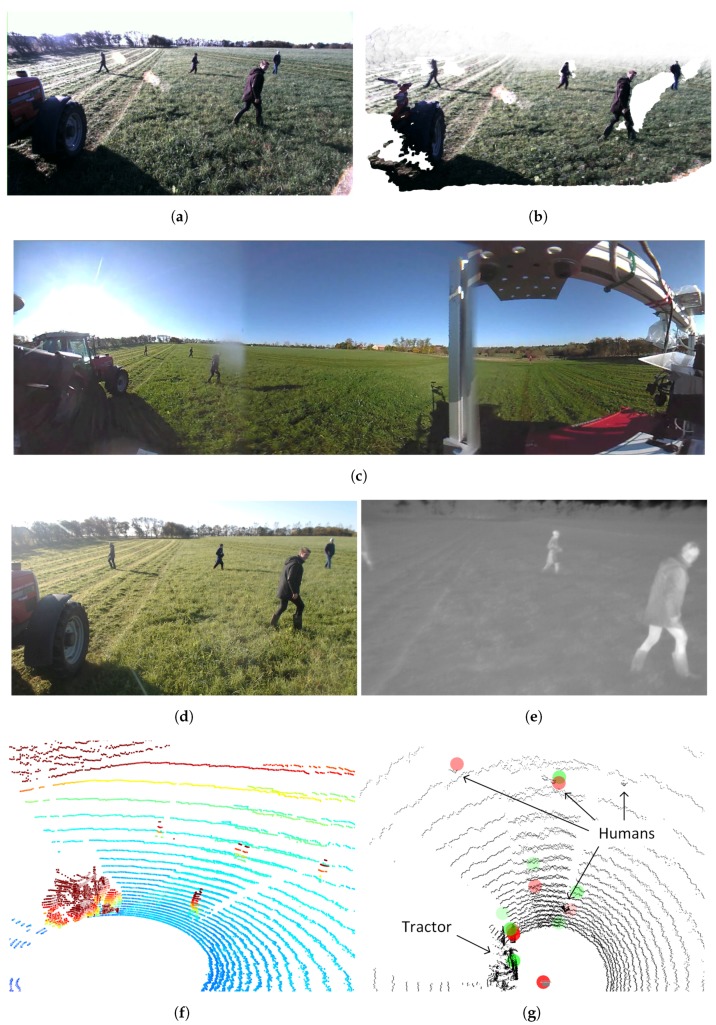
Example frames from the FieldSAFE dataset. (**a**) Left stereo image; (**b**) stereo pointcloud; (**c**) 360${}^{\circ }$ camera image (cropped); (**d**) web camera image; (**e**) thermal camera image (cropped); (**f**) LiDAR point cloud (cropped and colored by height); (**g**) radar detections overlaid on LiDAR point cloud (black). Green and red circles denote detections from mid- and long-range modes, respectively.

Figure 3 illustrates a synchronized pair of frames from stereo camera, 360${}^{\circ }$ camera, web camera, thermal camera, LiDAR and radar. 

Synchronization: Trigger signals for the stereo and thermal cameras were synchronized and generated from a pulse-per-second signal from an internal GNSS in the LiDAR, which allowed exact timestamps for all three sensors. The remaining sensors were synchronized in software using a best-effort approach in ROS, where the ROS system time was used to timestamp each message once it got delivered. However, best-effort message delivery does not provide any guarantees for delivery times, and the specific time delays for the different sensors therefore depend on the internal processing in the sensor, the transmission to the computer, network traffic load, the kernel scheduler and software drivers in ROS [33]. Time delays can therefore vary significantly and are not necessarily constant.

IMU and GNSS both use serial communication and therefore have very small transmission latencies. The same applies for radar that sends its data on the CAN bus. The web camera, however, uses a USB 2.0 interface and thus experiences a short delay in the transmission. A typical delay for the web camera has been measured as 100 ms. The 360${}^{\circ }$ camera uses the TCP protocol and experiences a large amount of packet retransmissions. The delay has therefore been measured up to 4.5 s. The time delays are both specified in relation to the stereo camera, which is synchronized to the LiDAR and thermal camera. 

**Figure 4:**
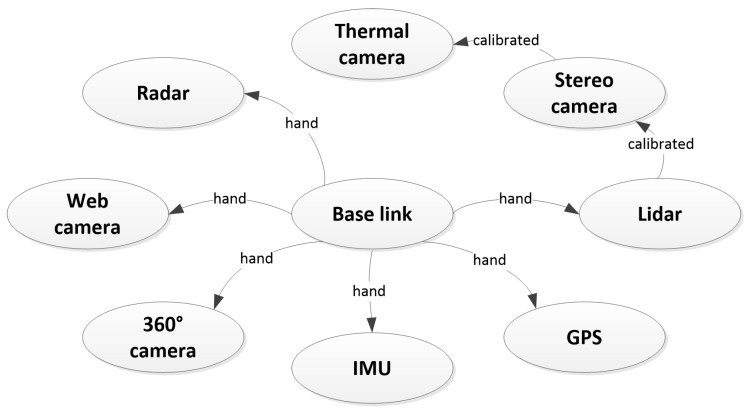
Sensor registration. “Hand” denotes a manual measurement by hand, whereas “calibrated” indicates that an automated calibration procedure was used to estimate the extrinsic parameters.

Registration: All sensors were registered by estimating extrinsic parameters (translation and rotation). A common reference frame, base link, was defined at the mount point of the recording frame on the tractor. From here, extrinsic parameters were estimated either by hand measurements or using automated calibration procedures. Figure 4 illustrates the chain of registrations and how they were carried out. The LiDAR and the stereo camera were registered by optimizing the alignment of 3D point clouds from both sensors. For this procedure, the iterative closest point (ICP) was used on multiple static scenes. An average over all scenes was used as the final estimate. The stereo and thermal cameras were registered and calibrated using the camera calibration method available in the Computer Vision System Toolbox in MATLAB. Since the thermal camera did not perceive light in the visual spectrum, a custom-made visual-thermal checkerboard was used. For a more detailed description of this procedure, we refer the reader to [34]. The remaining sensors were registered by hand, by estimating extrinsic parameters of their positions. All extrinsic parameters are contained in the dataset. Instructions for how to extract these are available at the FieldSAFE website. Here, the estimated intrinsic camera parameters are further available for download.

## 3. Dataset

**Figure 5:**
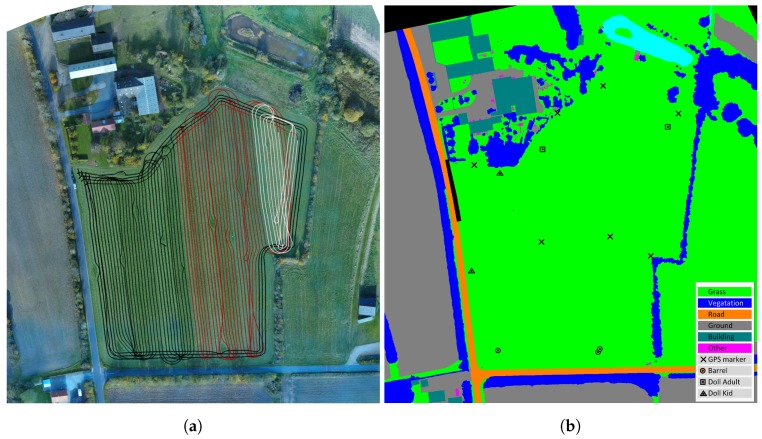
Colored and labeled orthophotos. (**a**) Orthophoto with tractor tracks overlaid. Black tracks include only static obstacles, whereas red and white tracks also have moving obstacles. Currently, red tracks have no ground truth for moving obstacles annotated. (**b**) Labeled orthophoto.

The dataset consists of approximately 2 h of recordings during grass mowing in Denmark, 25 October 2016. The exact position of the field was 56.066742, 8.386255 (latitude, longitude). Figure 5a shows a map of the field with tractor paths overlaid. The field is 3.3 ha and surrounded by roads, shelterbelts and a private property.

**Figure 6:**
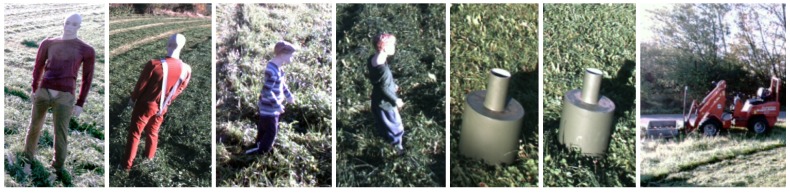
Examples of static obstacles.

A number of static obstacles exemplified in Figure 6 were placed on the field prior to recording. They included mannequin dolls (adults and children), rocks, barrels, buildings, vehicles and vegetation. Figure 5b shows the placement of static obstacles on the field overlaid on a ground truth map colored by object classes.

**Figure 7:**
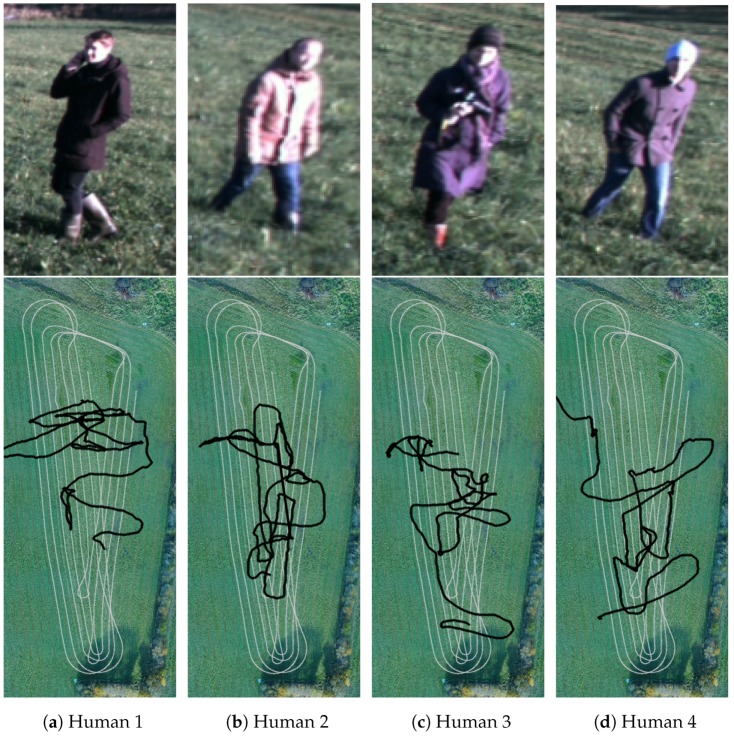
Examples of moving obstacles (from the stereo camera) and their paths (black) overlaid on the tractor path (grey).

Additionally, a session with moving obstacles was recorded where four humans were told to walk in random patterns. Figure 7 shows the four subjects and their respective paths on a subset of the field. The subset corresponds to the white tractor tracks in Figure 5a. The humans crossed the path of the tractor a number of times, thus emulating dangerous situations that must be detected by a safety system. Along the way, various poses such as standing, sitting and lying were represented.

During the entire traversal and mowing of the field, data from all sensors were recorded. Along with video from a hovering drone, a static orthophoto from another drone and corresponding manually-annotated class labels, these are all available from the FieldSAFE website.

## 4. Ground Truth

Ground truth information on object location and class labels for both static and moving obstacles is available as timestamped global (geographic) coordinates. By transforming local sensor data from the tractor into global coordinates, a simple look-up of the class label in the annotated ground truth map is possible.

Prior to traversing and mowing the field, a number of custom-made markers were distributed on the ground and measured with exact global coordinates using a handheld Topcon GRS-1 RTK GNSS. A DJI Phantom 4 drone was used to take overlapping bird’s-eye view images of an area covering the field and its surroundings. Pix4D [35] was used to stitch the images and generate a high-resolution orthophoto (Figure 5a) with a ground sampling distance (GSD) of 2 cm. The orthophoto was manually labeled pixel-wise as either grass, ground, road, vegetation, building, GPS marker, barrel, human or other (Figure 5b). Using the GPS coordinates of the markers and their corresponding positions in the orthophoto, a mapping between GPS coordinates and pixel coordinates was estimated.

For annotating the location of moving obstacles, a DJI Matrice 100 was used to hover approximately 75 m above the ground while the tractor traversed the field. The drone recorded video at 25 fps with a resolution of 1920 × 1080. Due to limited battery capacity, the recording was split into two sessions of each 20 min. The videos were manually synchronized with sensor data from the tractor by introducing physical synchronization events in front of the tractor in the beginning and end of each session. Using the seven GPS markers that were visible within the field of view of the drone, the videos were stabilized and warped to a bird’s-eye view of a subset of the field. As described above for the static orthophoto, GPS coordinates of the markers and their corresponding positions in the videos were then used to generate a mapping between GPS coordinates and pixel coordinates. Finally, the moving obstacles were manually annotated in each frame of one of the videos using the vatic video annotation tool [36]. Figure 7 shows the path of each object overlaid on a subset of the orthophoto. The second video is yet to be annotated.

## 5. Summary and Future Work

In this paper, we have presented a calibrated and synchronized multi-modal dataset for obstacle detection in agriculture. The dataset supports research into object detection and classification, object tracking, sensor fusion, localization and mapping. We envision the dataset to facilitate a wide range of future research within autonomous agriculture and obstacle detection for farming vehicles.

In future work, we plan on annotating the remaining session with moving obstacles. Additionally, we would like to extend the dataset with more scenarios from various agricultural environments while widening the range of encountered illumination and weather conditions.

Currently, all annotations reside in a global coordinate system. Projecting these annotations to local sensor frames inevitably causes localization errors. Therefore, we would like to extend annotations with, e.g., object bounding boxes for each sensor.

yes References

## Figures and Tables

**Table 1:** Comparison to existing datasets in robotics and agriculture.

Dataset	Environment	Length	Localization	Sensors	Obstacles	Annotations
KITTI [22]	urban	6 h	✓	stereo camera, LiDAR	cars, trucks, trams, pedestrians, cyclists	2D + 3D bounding boxes
Oxford [20]	urban	1000 km	✓	stereo camera, LiDARs, color cameras	cars, trucks, pedestrians, cyclists	none
Marulan [29]	rural	2 h	✓	lasers, radar, color camera, infra-red camera	humans, box, poles, bricks, vegetation	none
NREC [30]	orchards	8 h	✓	stereo camera	humans, vegetation	bounding boxes (only humans)
FieldSAFE (ours)	grass field	2 h	✓	stereo camera, web camera, thermal camera, 360${}^{\circ }$ camera, LiDAR, radar	humans, mannequins, rocks, barrels, buildings, vehicles, vegetation	GPS position and labels

**Table 2:** Exteroceptive sensors.

Sensor	Model	Resolution	FOV	Range	Acquisition Rate
Stereo camera	Multisense S21 CMV2000	1024 × 544	85${}^{\circ }$× 50${}^{\circ }$	1.5–50 m	10 fps
Web camera	Logitech HD Pro C920	1920 × 1080	70${}^{\circ }$× 43${}^{\circ }$	-	20 fps
360 ${}^{\circ }$ camera	Giroptic 360cam	2048 × 833	360${}^{\circ }$ × 292${}^{\circ }$	-	30 fps
Thermal camera	Flir A65, 13 mm lens	640 × 512	45${}^{\circ }$ × 37${}^{\circ }$	-	30 fps
LiDAR	Velodyne HDL-32E	2172 × 32	360${}^{\circ }$ × 40${}^{\circ }$	1–100 m	10 fps
Radar	Delphi ESR	16 targets/frame	90${}^{\circ }$ × 4.2${}^{\circ }$	0–60 m	20 fps
		16 targets/frame	20${}^{\circ }$ × 4.2${}^{\circ }$	0–174 m	20 fps

**Table 3:** Proprioceptive sensors.

Sensor	Model	Description	Acquisition Rate
GPS	Trimble BD982 GNSS	Dual antenna RTK GNSS system. Measures position and horizontal heading of the platform.	20 Hz
IMU	Vectornav VN-100	Measures acceleration, angular velocity, magnetic field and barometric pressure.	50 Hz
